# Conservative Management of a Congenital Seminal Vesicle Cyst Associated with Ipsilateral Renal Agenesis Revealed by Cystitis: One Case Report

**DOI:** 10.1155/2011/125753

**Published:** 2011-11-21

**Authors:** Youness Ahallal, Mohammed Fadl Tazi, Abdelhak Khallouk, Jalaleddine Elammari, Mohammed Jamal Elfassi, Moulay Hassan Farih

**Affiliations:** Department of Urology, Hassan II Teaching Hospital, Fes, Morocco

## Abstract

Seminal vesicle cyst is an extremely rare disease. Its association with ipsilateral renal agenesis is even more exceptional. We present herein one case of a 16-year-old male who presented with a four-month history of lower urinary tract symptoms (LUTSs) and micturition pain. The digital rectal examination revealed a small mass arising from the prostate. The urine culture showed that *E. coli* is sensitive to all antibiotics tested. Transrectal ultrasound (TRUS) revealed a cystic mass in the outer prostate. Seminal vesicle cyst and left renal agenesis were confirmed by magnetic resonance imaging (MRI). Maximum flow (*Q*_max_) at uroflow was greater than 15 mL/sec. We therefore decided to manage this disease conservatively with alpha blockers and antibiotics. After 6-month' followup the patient did not report any complain and the uroflow test was similar to a normal urination. From one case report and literature review, the authors suggest a diagnostic and therapeutic strategy for the management of this rare condition.

## 1. Introduction

A seminal vesicle cyst in combination with ipsilateral renal agenesis is rarely encountered with fewer than 100 cases reported in the literature [1, 2]. Treatment of the seminal vesicle cyst can be decided according to symptom existence. Many authors report surgical treatment of seminal vesicle cyst but surgery is very demanding and features many complications because of the deep location and dissection difficulty of the seminal vesicles in the retrovesical space.

We present one case of seminal vesicle cyst with ipsilateral renal agenesis, who was successfully treated conservatively with alpha-blockers.

## 2. Case presentation

**Figure 1:**
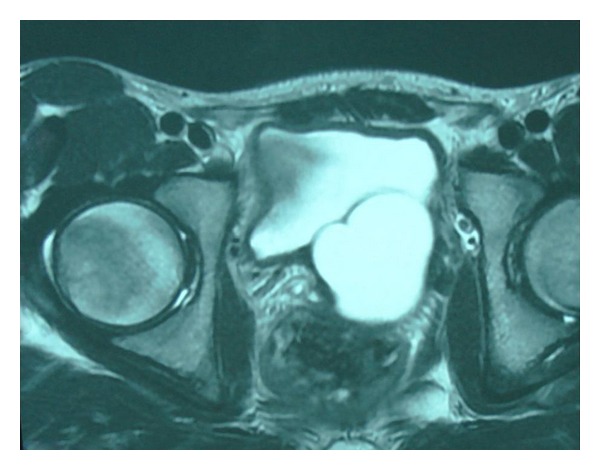
Pelvic MRI showing a seminal vesicle cyst.

**Figure 2:**
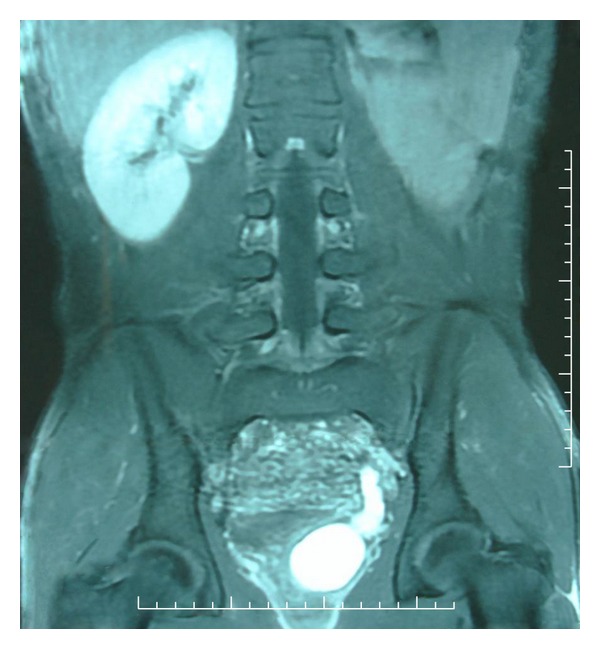
MRI showing a tube-like structure meandering along the rear wall of the bladder corresponding to the distal ureter, a cyst in the seminal vesicle area and renal agenesis.

A 16-year-old Moroccan male presented with a four-month history of LUTS and micturition pain. The digital rectal examination revealed a large, soft, fluctuant mass arising from the prostate. TRUS revealed a cystic mass measuring 5 × 4 × 3.5 cm arising from the prostate and no left kidney was seen. On MRI, a tube-like structure meandering along the rear wall of the bladder was recognized as the lower portion of the left ureter. The mass showed low T1- and high T2-weighted signal intensity compatible with a cyst in the left seminal vesicle area (Figures 1 and 2). Enhanced CT revealed left renal agenesis and a retroperitoneal cystic lesion. The urine culture showed that *E. coli* was sensitive to all antibiotics tested and *Q *_max_ at uroflow was greater than 15 mL/second. As surgery is very demanding and features many complications, we decided to manage this disease conservatively with alpha-blockers and antibiotics. After 6-month followup, the patient did not report any complain and the uroflow test was similar to a normal urination.

## 3. Discussion

First reported in 1872 by Smith NR [2], cysts of the seminal vesicles associated with renal agenesis are extremely rare with fewer than 100 cases reported in the English literature [3]. During renal sonographic mass screening among 280,000 children in a 2.5-year period in Taipei, the reported incidence of cystic dilatations in the pelvis with ipsilateral renal agenesis or dysplasia was 0.0046% [1].

Most of them are congenital cause; most authors believe it to be secondary to obstruction of the ejaculatory duct. It is associated with anomalies of maldevelopment of the distal portion of the mesonephric duct, such as renal dysplasia, renal agenesis, and ectopic ureter into the seminal vesicle [4].

The majority of seminal vesicle cysts are small (<5 cm). Most of them are asymptomatic. The clinical presentation is either dysuria or urinary tract infection [4]. The cyst can grow and irritate the surrounding viscera, resulting in lower urinary tract symptoms, suprapubic pain, hematospermia, and painful ejaculation [1].

Applicable diagnostic methods include digital rectal examination, excretory urography, TRUS, CT, MRI, and cystoscopy. [1] Digital rectal examination usually shows a palpable fluctuant mass arising from the superior aspect of the prostate gland [3].

Sonographic findings can confirm the cystic nature of the pelvic mass and determine the relative size and location. Findings include an anechoic pelvic mass with a thick, irregular wall and occasional wall calcifications, or the mass may contain internal debris reflecting prior hemorrhage or infection [5].

Abdominal computed tomography, magnetic resonance imaging, and seminal vesiculography are useful for the detection of accompanying deformity and differential diagnosis. A cystoscopy helps to confirm a hemitrigone, absence of ureteral orifice and other anomalies in bladder [1].

If the condition is asymptomatic, observation without treatment is the norm, but surgical resection, transurethral resection, cyst puncture, and so forth may be performed in patients with symptoms [6]. Recently, laparoscopic surgery has appeared to be most suitable for surgical treatment of seminal vesicle cyst as it features the advantage of minimal invasiveness and direct access to the seminal vesicle area with an excellent image compared to open surgery [1].

Surgical management would improve subjective symptoms; however it remains uncertain whether it would improve the patient's sperm properties as well [7]. Besides, since surgery is also associated with high complications rate (including damage of bladder and rectum, sexual dysfunction), we first decided to treat our patient using alpha-blockers with a regular observation. This strategy seems to be efficient as the patient was asymptomatic after 3 months of treatment.
